# Online trade of wild game meat: Implications for public health and conservation

**DOI:** 10.1007/s13280-025-02221-w

**Published:** 2025-07-24

**Authors:** Tomohiko Endo, Kota Mameno, Takahiro Kubo

**Affiliations:** 1 Biodiversity Division, National Institute for Environmental Studies, 16-2 Onogawa, Tsukuba, Ibaraki 305-8506 Japan; 2 Institute of Livestock and Grassland Science, National Agriculture and Food Research Organization (NARO), 2-1-18 Kannondai, Tsukuba, Ibaraki 305-8666 Japan; 3 Research Faculty of Agriculture, Hokkaido University, Kita 9 Nishi 9, Kita-Ku, Sapporo, 060-8589 Japan; 4 Graduate School of Agriculture, Hokkaido University, Kita 9 Nishi 9, Kita-Ku, Sapporo, 060-8589 Japan; 5 School of Geography and the Environment, University of Oxford, S Parks Rd, Oxford, OX1 3QY UK

**Keywords:** Consumer preference, Human–wildlife conflict, One health, Online auction, Wild game meat

## Abstract

Trade in wild game meat (WGM) is increasing in online markets. However, the distribution of WGM in online trade remains unclear, and our limited knowledge has prevented sustainable WGM consumption that reduces the risks to human health and overcomes the challenges in wildlife management and biodiversity conservation. To explore the trends and challenges of online transactions of WGM, we analyzed the sales data from the online consumer-to-consumer market in Japan. Results revealed a high propensity to trade the lean meat. Our results also showed that transactions of edible offal were significantly positive and relatively more common in wild boar and black bear. In the brown bear, the coefficient for the sales numbers of “hand and foot” was significantly positive. Findings indicate that the online consumer-to-consumer market can be a new opportunity to distribute WGM. The development of guidelines and regulatory systems could help to avoid the risks associated with WGM consumption.

**Supplementary Information:**

The online version contains supplementary material available at 10.1007/s13280-025-02221-w.

## Introduction

Wild meat has an important role in food security, cultural traditions, and local economies (Milner-Gulland et al. 2003; Ingram et al. 2021), while it has several risks. In parts of Southeast Asia, Africa, and South America, wild meat (often referred to as bushmeat) is an important source of food and income for rural communities, and it makes a significant contribution to food security and socio-economic stability (Nasi et al. 2008; Lindsey et al. 2013). On the other hand, there are proven public health risks, such as the spread of emerging infectious diseases and zoonoses (Ingram et al. 2021; van Vliet et al. 2022). Moreover, unsustainable wild meat consumption increases biodiversity conservation risks, such as wildlife extinction and degradation of ecosystems and their services; increased wild meat demand also poses a serious threat to biodiversity conservation by encouraging illegal wildlife trade (Ripple et al. 2016; McEvoy et al. 2019). Hence, quantitative assessment of trade dynamics is essential for wild meat use to balance between risks and benefits, such as biodiversity conservation, public health, wildlife management, and socioeconomics (Pruvot et al. 2019).

In developed countries, wild meat harvested legally or under a regulatory framework, often referred to as wild game meat (WGM), is consumed for a variety of purposes (Ripple et al. 2016; Needham et al. 2023). The consumption of WGM has increased in recent years because of several real and perceived benefits. Consuming WGM, for example, can not only improve people’s health and reduce the environmental impacts of farming, but can also help with wildlife management (Marescotti et al. 2019; Corradini et al. 2022). Well-managed WGM can be a healthy food resource because it is lower in fat and cholesterol than livestock meat and contains more high-quality protein, minerals, and amino acids (Czarniecka-Skubina et al. 2022; Needham et al. 2023). WGM consumption has a lower environmental impact than livestock because of reduced greenhouse gas emissions and water pollution (Marescotti et al. 2019; Czarniecka-Skubina et al. 2022). Furthermore, the expanded consumption of harvested WGM is expected to improve wildlife management through increasing hunting incentives and sustainable resource uses (Ljung et al. 2012; Needham et al. 2023), that leads to sustainable and thoughtfully planned management strategies mitigating human–wildlife conflict. Indeed, the Japanese government, for example, promotes the WGM uses (Ministry of Agriculture and Forestry and Fisheries (MAFF), 2025a).

With growing demands and developing technology, WGM has reverently been traded through various platforms including online markets. Yet, WGM consumption through the unestablished markets has challenges not only for human safety and public health but also for biodiversity conservation (Czarniecka-Skubina et al. 2022). WGM is generally traded in both formal and informal markets after being caught and divided into carcasses and offal; thus, the WGM distribution process creates a risk of exposure to foodborne and other pathogens (Hedman et al. 2020). Previous reports noted risk of food poisoning due to zoonosis and bacterial contamination (Hedman et al. 2020; Niewiadomska et al. 2021) and lead poisoning from bullets (Pain et al. 2010; Green and Pain 2019). Therefore, WGM trade for consumption requires processing in facilities that comply with Hazard Analysis and Critical Control Point hygiene standards and the establishment of a meat distribution system that guarantees traceability (Ramanzin et al. 2010; Needham et al. 2023). The recent growing popularity of online trade has the potential to exacerbate public health and biosecurity risks by increasing the volume of sales and broadening the distribution of wildlife products (Moloney et al. 2023). The online trade of WGM can also provide negative impacts on biodiversity conservation. For instance, online trade is used as a convenient platform for promoting the sales of wild animals, and it could facilitate the trade of illegal or unregulated harvested wildlife through unmonitored supply chains (Moloney et al. 2023). Similarly, increasing the WGM demand on the online market could cause animal welfare issues through cruel hunting/trapping methods. Trade of bear parts is sometimes led by criminal and/or highly organized networks, which contributes to the strengthening of illicit economies and poses serious conservation risks (Nijman et al. 2017). However, despite the inherent risks associated with the online WGM trade, little is known about the distribution status of WGM in online trade. The limited knowledge has prevented the assessment of the human health risk and challenges for wildlife management and biodiversity conservation of WGM consumption.

To address this knowledge gap, we analyzed online WGM trade data by focusing on the Japanese online consumer-to-consumer (CtoC) markets. Our main objectives were to reveal the sales trends of large mammal WGM in online markets and to discuss the challenges to sustainable WGM consumption based on the evidence of the current distribution on the online market. We used the online sales data on one of the biggest and long-standing online CtoC platforms in Japan, Yahoo Auction^1^ although WGM is tradable in other CtoC platforms in Japan (e.g., mercari:^2^; jimoty:^3^). The platform allows people to buy and sell a variety of wildlife-related products from living organisms to meat, specimens, and even pest control products, and the annual transaction volume exceeds 10 trillion JPY (around USD 66.67 billion). The large-volume objective sales information can outline WGM trade in Japan and provides helpful insights for the management. Given outbreaks of zoonotic and foodborne bacterial and parasitic infection in WGM have been reported in Japan (Sasaki et al. 2013; Kobayashi et al. 2021), understanding the trends of online sales of WGM can help to develop sustainable WGM consumption. Furthermore, this study makes several important contributions by determining the distribution of the CtoC online market. The present study would contribute to wildlife management for human–wildlife conflict mitigation and healthier ecosystems. Given the insufficient effort of wildlife management particularly in rural areas under depopulation (Tsunoda and Enari 2020), understanding WGM’s market demand is necessary, as it can be a potential driver of culling and hunting by the market mechanism. While nonlethal conflict mitigation remains important, our research on WGM trade resulting from culling and hunting provides critical insights for the development of effective wildlife management policies. Finally, by exploring public health challenges posed by WGM consumption, this study also aims to contribute to the development of “One Health” approach for the sustainable health of animals, humans, and the environment (van Vliet et al. 2016; Hedman et al. 2020; Needham et al. 2023).

### Background of human–wildlife conflict and WGM consumption in Japan

**Fig. 1:**
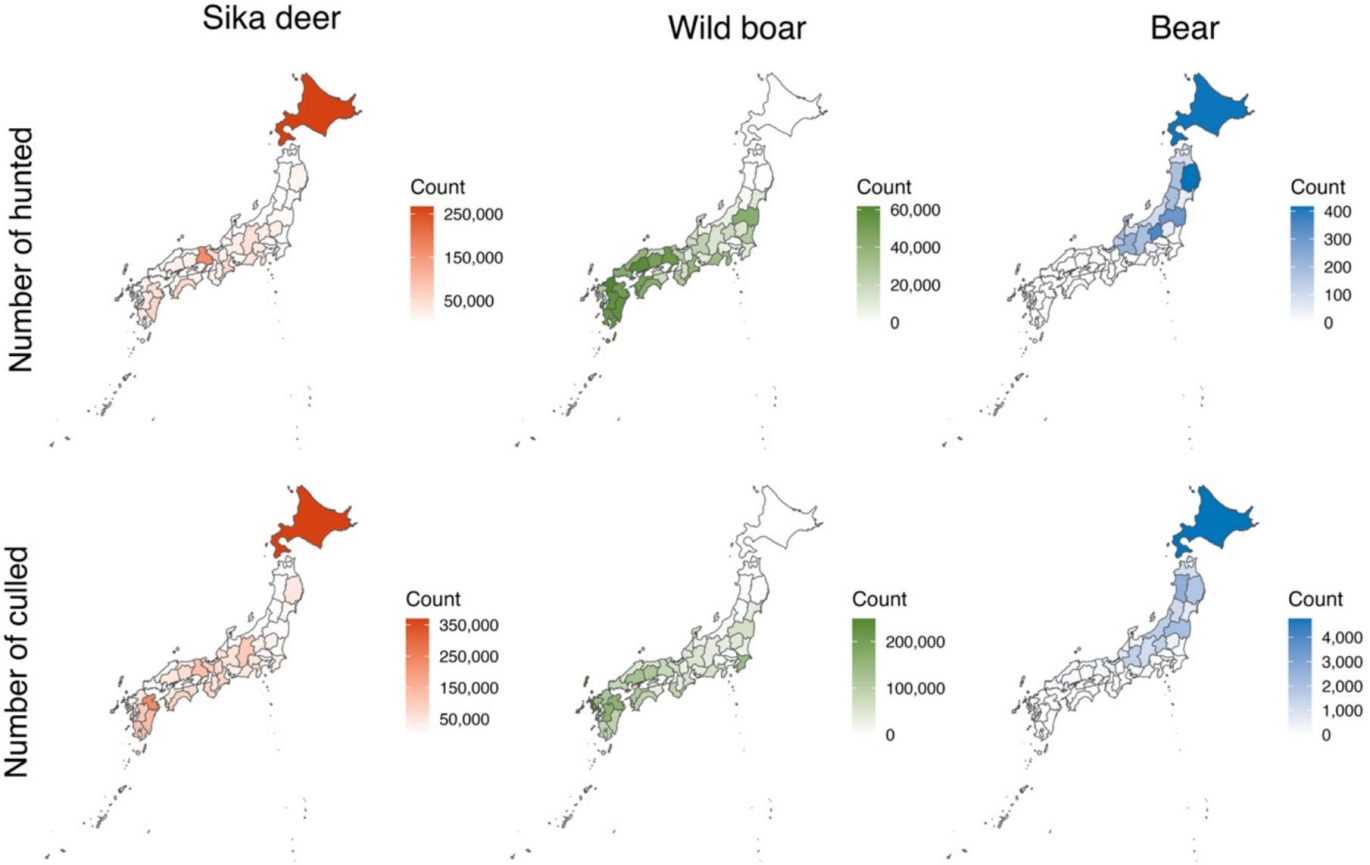
Numbers of large wildlife hunted and culled nationwide in Japan from FY 2013 to 2019. Note: (1) Data source is the Ministry of the Environment (2019). (2) Bears include Asiatic black bears and brown bears

Human–wildlife conflict is a major challenge in biodiversity conservation, public health, and the local economy in Japan. That is caused by various factors, including reduced snowfall due to climate change, the aging of farmers and hunters, and decreased human activity due to population decline, making it a particularly serious problem in rural areas (Tsunoda and Enari 2020; Iijima et al. 2023; Morosawa et al. 2024). Some effective measures including both lethal and nonlethal approaches have been introduced to mitigate human–wildlife conflicts. Of them, the Japanese government promotes culling and hunting, as a result, the sika deer and wild boar are heavily harvested (Tsunoda and Enari 2020; Okuda et al. 2022). Recently, approximately 700 000 sika deer and 600 000 wild boar have been harvested through recreational hunting and government-mandated culling each year (MoE 2019; Ministry of the Environment (MoE) 2024; Fig. 1). Regarding bears, there are two species living in Japan: the brown bear (*Ursus arctos yesoensis*) (in Hokkaido only) and the Asiatic black bear (*Ursus thibetanus*) (in Honshu and Shikoku) (Sato 2017). In recent years, the number of conflicts in urban areas and human injuries caused by bears has been increasing, prompting the implementation of nationwide measures to address the issue (MoE 2024). For these four species, hunters with a hunting license are permitted to hunt during the hunting season. The hunting season is from October to January in Hokkaido, and from November to February in other regions. Furthermore, in recent years, culling has been carried out throughout the year by people (mainly hunters) who have obtained special permission from the prefecture for the purpose of nuisance and population control (MoE 2025).

Some of the harvested wildlife are consumed as WGM; moreover, WGM consumption has increased in recent years in Japan (Sasaki et al. 2022). In Japan, WGM tends to be consumed for social benefits and consumer utility rather than for subsistence (Kadohira et al. 2019). In 2023, the number of wild game animals processed at licensed slaughterhouses was 121 117 deers, 39 918 wild boars, and 664 bears including both Asiatic black bears and brown bears. The amount of WGM traded for consumption was 1184 tons for deers, 511 tons for wild boars, and 11 tons for bears including both Asiatic black bears and brown bears (MAFF 2024a). In Japan, health risks associated with WGM consumption have been noted, such as the risk of hepatitis E virus infection from consuming sika deer or wild boar meat and the risk of trichinellosis from consuming bear meat (Takahashi et al. 2004; Murakami et al. 2023). On the other hand, the consumption of WGM has also been encouraged in Japan (MAFF 2025a). In Japan, the “Guidelines on the hygienic management of wild meat” were established in 2014 to ensure hygiene management from hunting to sales (Ministry of Health, Labor and Welfare (MHLW) 2014; (Takai 2022). Furthermore, in June 2020 the Food Sanitation Act was amended, and Hazard Analysis and Critical Control Point-based hygiene management has been mandatory since June 2021 (MHLW 2025). Currently, only WGM processed at special slaughterhouses licensed by the prefecture is permitted for sale. In addition, due to the outbreak of classical swine fever (CSF) in Japan from September 2018, the distribution of wild boar meat from areas where CSF is prevalent has been restricted since April 2021 (MAFF 2025b).

## Materials and methods

### Data collection and trimming

**Fig. 2:**
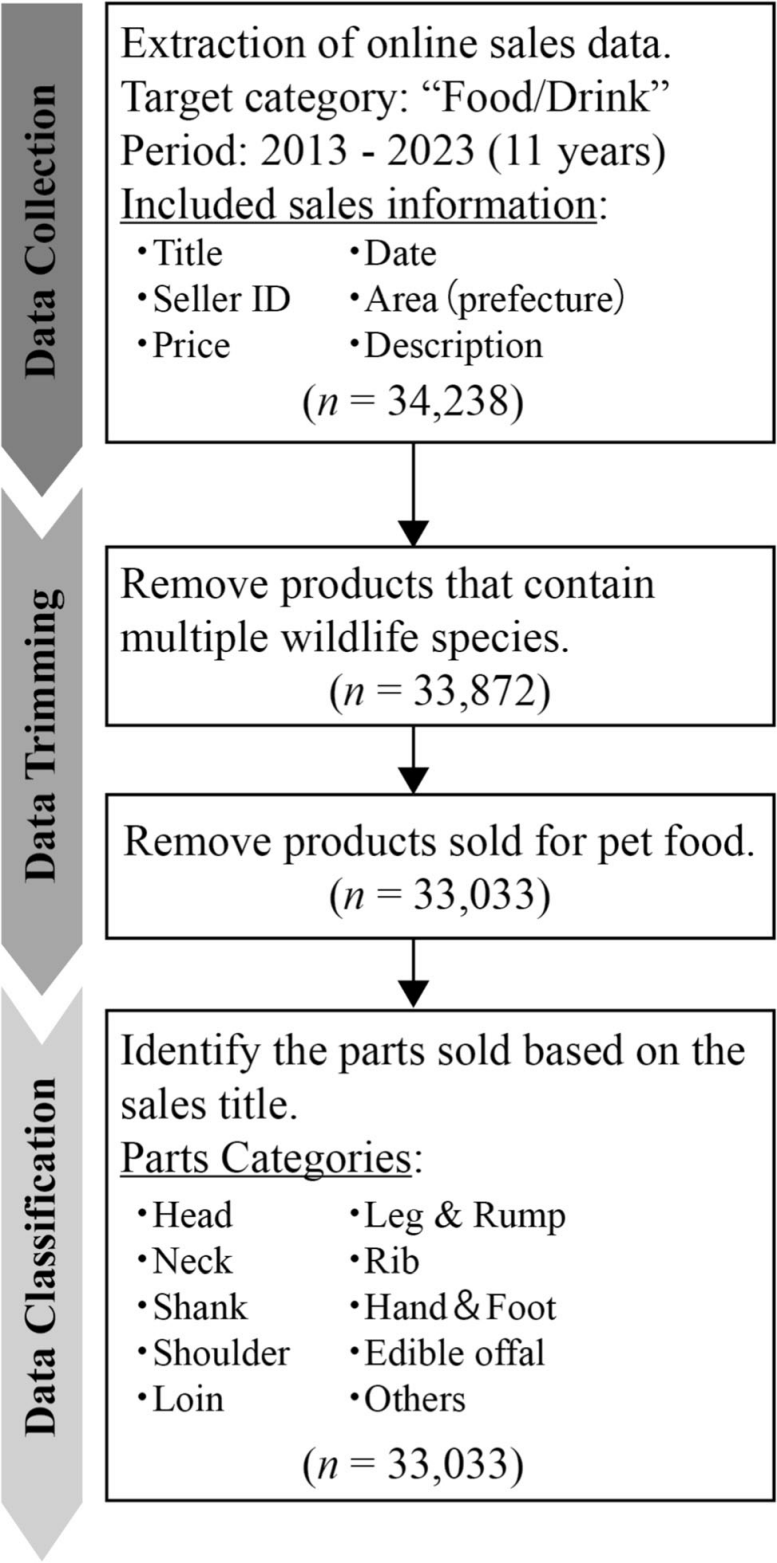
Flowchart of the selection process of online WGM sales data

We used sales data from Yahoo Auction (4), one of the largest online marketplaces in Japan, from January 2013 to December 2023 (11 years). The data selection process is shown in Fig. 2. The sales data were downloaded from Aucfan, a mirror website that archives the sales history of Yahoo Auction. The target category was “Food/Drink,” within which WGM is traded. The sales information extracted included the sale title, seller ID, sales price, sales date, sales area (prefecture), and sales description. To focus on WGM within the target category, we extracted all sales histories that contained the title “Sika deer meat” OR (“Gibie” AND “Sika deer”) OR “Yeso deer meat” OR (“Gibie” AND “Yeso deer”) OR “Wild boar meat” OR (“Gibie” AND “Wild boar”) OR “Asiatic black bear meat” OR (“Gibie” AND “Asiatic black bear”) “Brown bear meat” OR (“Gibie” AND “Brown bear”) OR “Bear meat” OR (“Gibie” AND “Bear”) (in Japanese).

We systematically detected species names from the title information to identify the species of WGM. This method accurately detects species names if they are included in the title text, so there is no possibility of species name misidentification. In Japan, there are multiple subspecies of sika deer and wild boar (e.g., Yeso deer, Ryukyu wild boar). In this study, we summarized at the species level and categorized them into four species: sika deer, wild boar, Asiatic black bear, and brown bear. About “Bear meat,” we identified bear meat from Hokkaido as brown bear and bear meat from other regions as Asiatic black bear, based on sales area information. The data also included set sales that mixed parts from multiple animals, as well as sales for pet foods. Here, our objective was to clarify the sales trends and consumer preferences of each wild animal as food. Therefore, we excluded from the datasets those sales where the relationship between animal species and meat parts was unclear (i.e., set sales), as well as sales for pet foods (Fig. 2); as a result, a total of 366 sales of set sales and 839 sales for pet foods were excluded. Since these are very small numbers in the total sales (set sales: 1%, pet use: 2%), they do not affect the results.

**Fig. 3:**
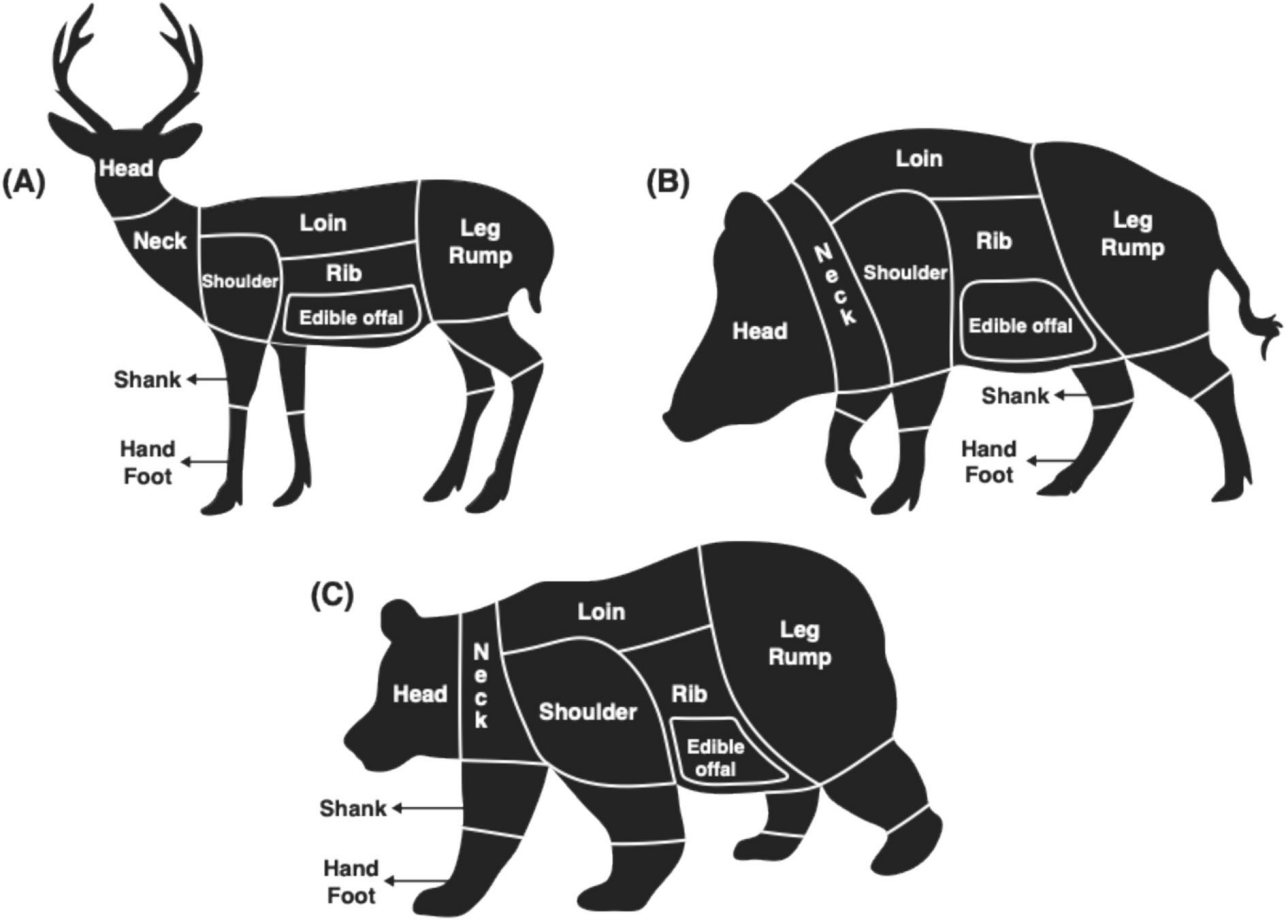
Image of meat cuts from wild animals used for consumption

We classified the body parts sold into 10 categories based on the domestic WGM cutting chart (MAFF 2024b): “Head,” “Neck,” “Shank,” “Shoulder,” “Loin,” “Leg & Rump,” “Rib,” “Hand & Foot,” “Edible offal,” and “Others” (Figs. 2 and 3). We recorded whether each item matched these categories as either 0 or 1. We added a category variable of hunting season (“Hunting season” OR “Non-hunting season”) on the basis of the sales data in order to consider the effects of the hunting season.

### Statistical analysis

To explore consumer preferences for WGM, we used generalized linear mixed models (GLMMs) to evaluate the number of sales and sales prices of each body part. All data were summarized monthly. Since there was no significant change in the number of sales before and after the Food Sanitation Act amendment, all data were used for analysis (Fig. S1).

In the analysis of the number of sales, the response variable was the number of sales per month and the explanatory variable was the number of counts of each body part. We applied a log link function and a negative binomial distribution to the GLMMs, because the response variable showed a trend of overdispersion in a preliminary analysis that used the Poisson distribution. In the analysis of sales prices, the response variable was the average price per month and the explanatory variable was the number of counts of each body part. The response and explanatory variables were standardized with a mean of 0 and a standard deviation of 1 before we conducted the analysis. The response variable was used with a Gaussian distribution. In all models, to account for the effects of regionality and seasonality, we included the sales area and hunting season as random intercepts in the models.

We evaluated the multicollinearity between the explanatory variables based on the variance inflation factor, and we determined that there was no effect of multicollinearity when the variance inflation factor was < 3 (Zuur et al. 2010). We analyzed each species (i.e., number of sales, four models; sales price, four models; total eight models), and assessed the statistical significance of the regression coefficients for each explanatory variable by Wald test (*P* < 0.05). The analysis was conducted with the glmmTMB package (Brooks et al. 2017) in R v. 4.3.2 software.

## Results

### Trends in purchasing behavior

**Fig. 4:**
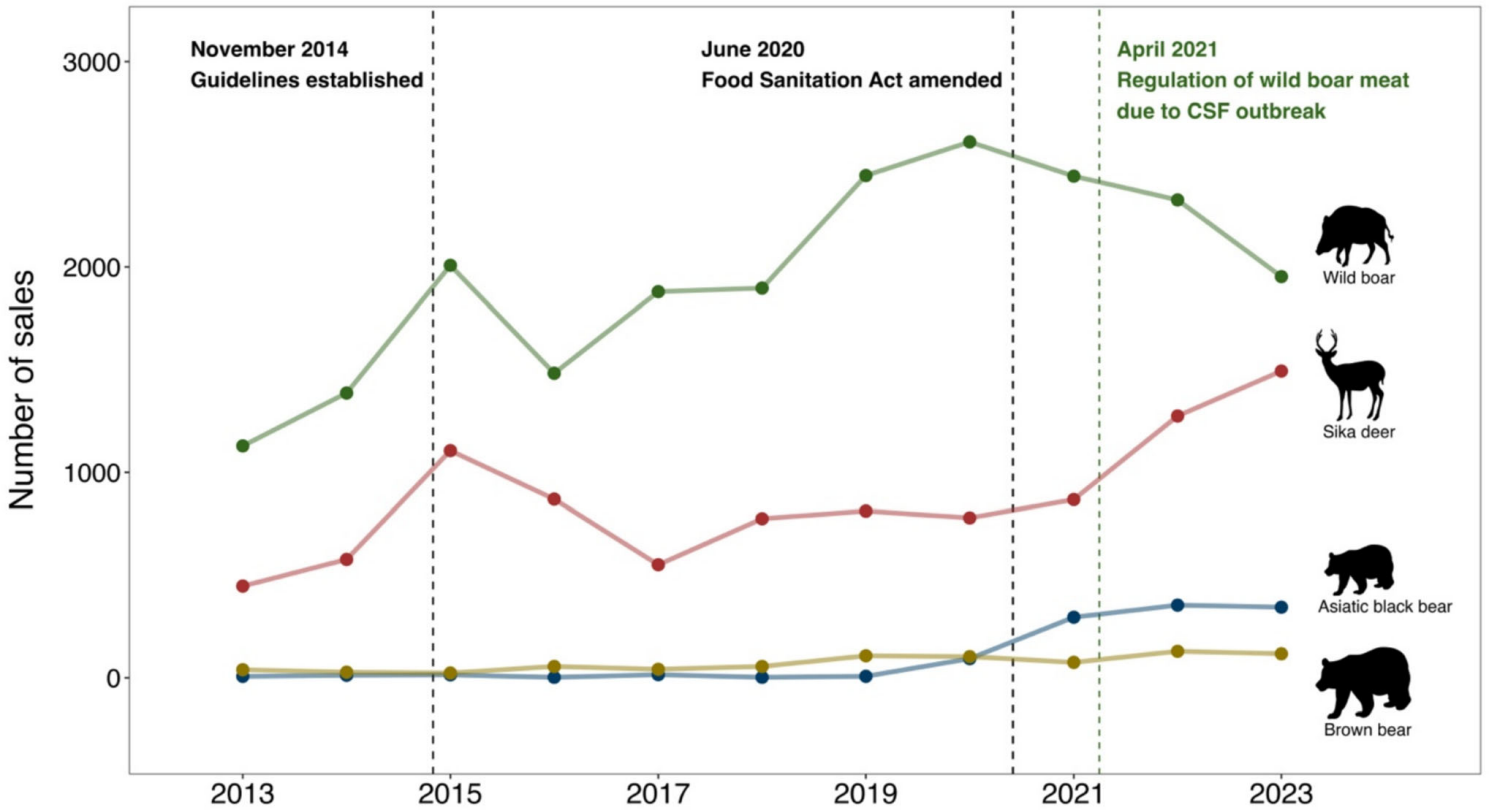
Trends in the total annual sales of each type of wild game meat. Note: (1) The black dashed line indicates the period when the WGM guidelines were established (left side) and when the Food Sanitation Act was amended (right side). (2) The green dashed line indicates the period when the distribution of wild boar meat was regulated due to CSF outbreaks

**Fig. 5:**
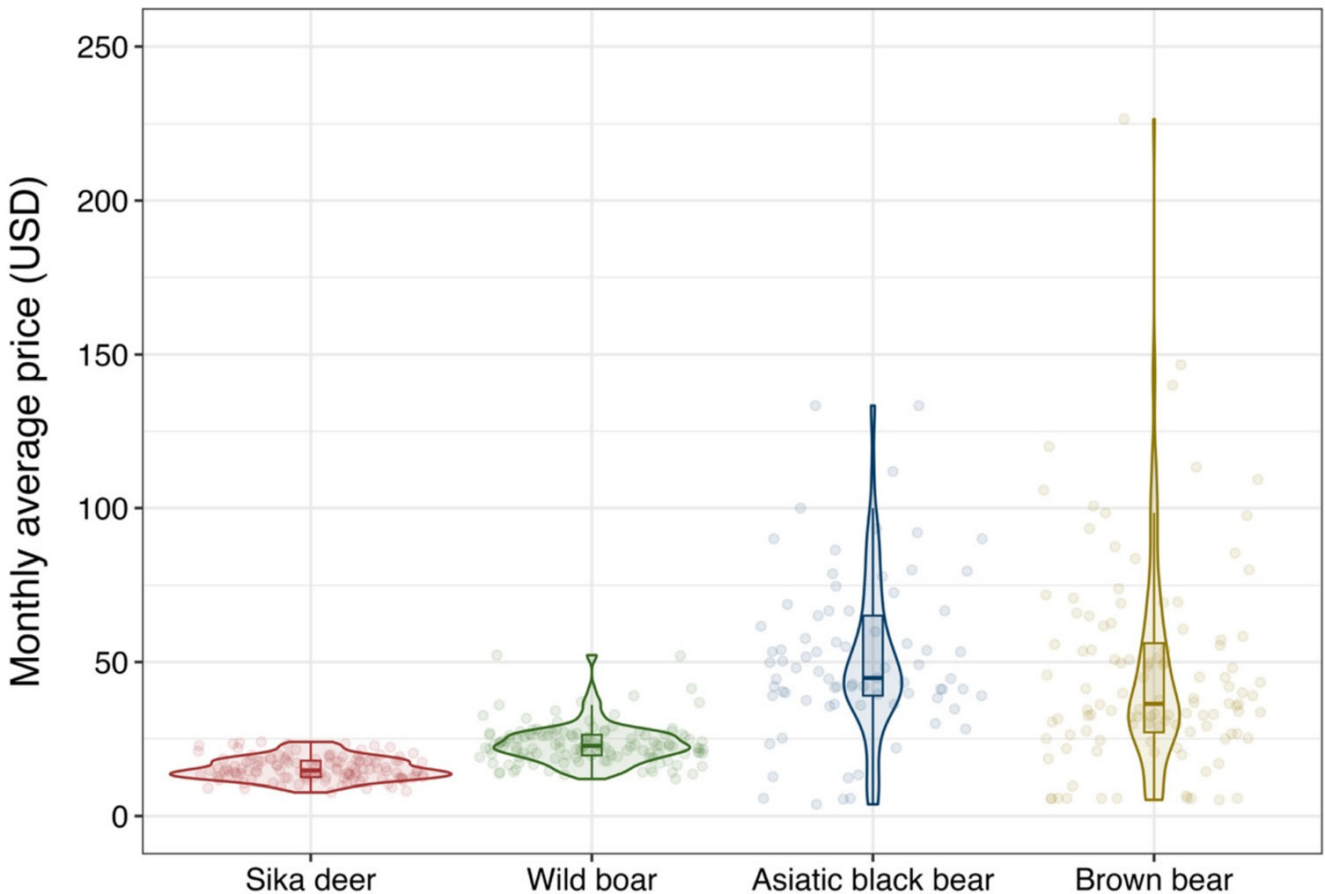
Monthly average price of each type of wild game meat. Note: (1) The violin plot indicates the frequency distribution of the data point. (2) The box plot within each violin plot indicates the median values (thick line), and the quartiles (25% and 75%) are represented by boxes with error bars

During the study period, a total of 33 033 WGM sales were traded. Wild boar had the most trades, with 21 563 transactions, and brown bears had the fewest, with 771 (Supplementary information on the volume is shown in Fig. S2). The number of trades of sika deer increased over the past four years, and in Asiatic black bears over the past three years. The number of trades of wild boar decreased from 2020, and the number of trades of brown bears was stable (Fig. 4). Asiatic black bears and brown bears were traded at higher prices than sika deer and wild boar (mean ± SD: USD 51.5 ± 26.3 and USD 46.2 ± 33.7) (calculated at 150 JPY = 1 USD, Fig. 5).

### Consumer preferences for WGM

**Fig. 6:**
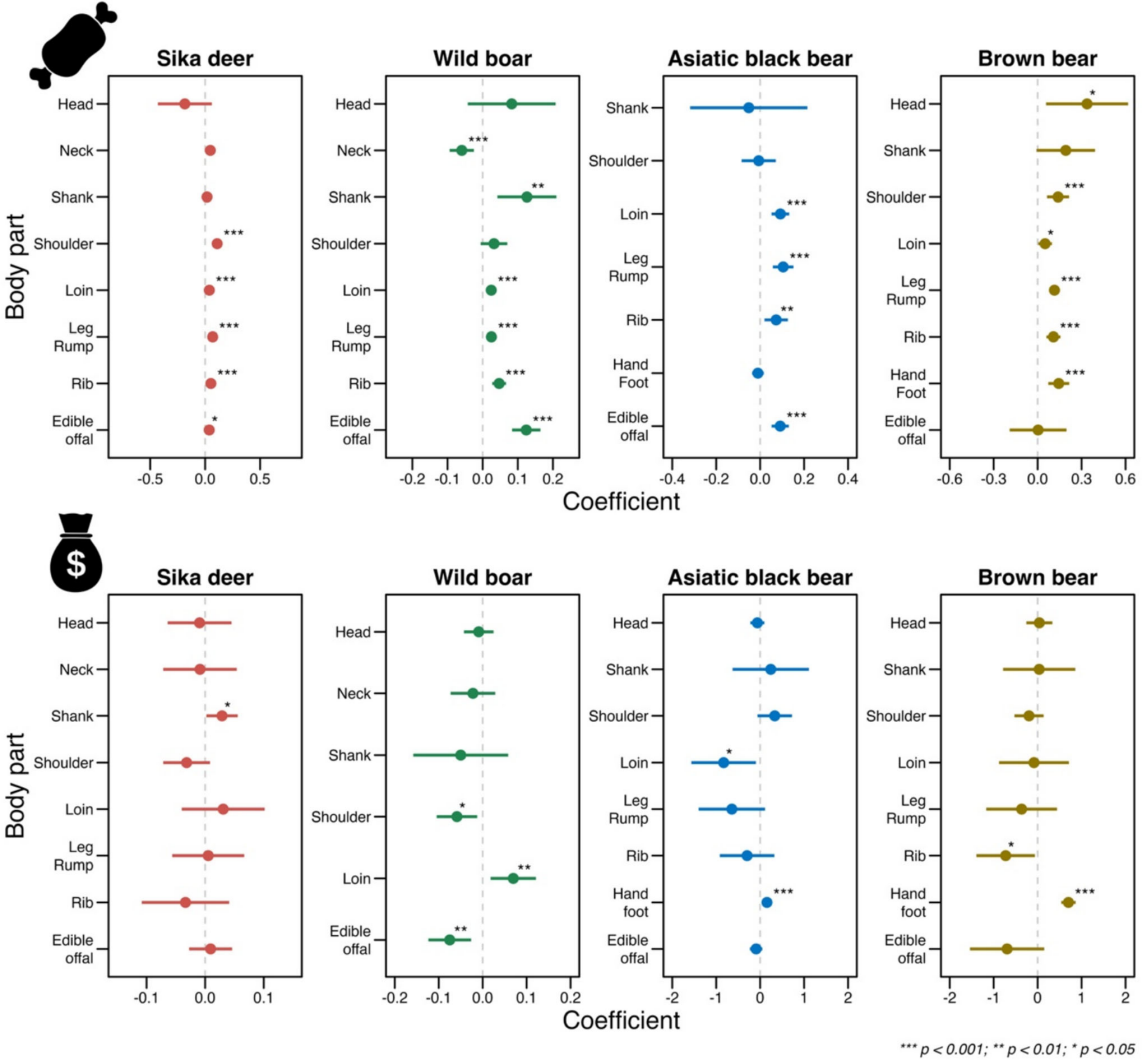
Estimated results of the generalized linear mixed model for the number of sales and price of each body part. Note: (1) The top panels show the results for the number of sales, and the bottom panels show the results for price. (2) High VIF variables were excluded from each model

The GLMMs for transaction volume showed that the coefficients for Loin, Leg & Rump, and Rib were significantly positive in all species (Fig. 6). This means that there was a strong trade in these major meat parts of all species (Table S1). Edible offal transactions were strongly significantly positive in wild boar (coefficient = 0.124, *z* = 6.024) and Asiatic black bear (coefficient = 0.091, *z* = 4.565). In wild boar, the Liver and Gallbladder were sold in large numbers. In Asiatic black bears, the Heart and Liver were frequently sold, while no sales of Gallbladders were observed. Details of the Edible offal sold are provided in Table S2. In the case of brown bear transaction volumes, the coefficient for Hand & Foot was significantly positive (coefficient = 0.144, *z* = 3.929). Detailed results of the model can be found in Table S3.

In our analysis of sale price, the coefficient values showed that sika deer Shank and wild boar Loin were traded at significantly high prices (sika deer: coefficient = 0.029, *z* = 2.110; wild boar: coefficient = 0.070, *z* = –2.647). Wild boar Edible offal was traded at significantly low prices (coefficient = –0.075, *z* = –3.023) (Fig. 6). Bear Hand & Foot were traded at significantly high prices (Asiatic black bear: coefficient = 0.155, *z* = 4.513; Brown bear: coefficient = 0.701, *z* = 8.397). Detailed results of the model can be found in Table S4.

## Discussion

Understanding the status of the WGM market—specifically its distribution and consumer preferences—is crucial for achieving sustainable WGM consumption, as it helps mitigate the challenges of conservation and public health risks, while also enhancing the benefits of wildlife management. Here, we assessed consumer preferences for WGM on the basis of the distribution status and trade log of large WGM in the online CtoC market. The results showed a high number of trades of lean meat of all animal species. Edible offal from wild boar and Asiatic black bears was traded in high volumes, and the offal from wild boar sold for relatively low prices. In addition, the Hand & Foot of Asiatic black bears and brown bears were traded at high prices.

This study shows that the online CtoC market is creating a new opportunity for distributing harvested sika deer and wild boar meat. We found that sika deer and wild boar dominated the distribution in the online CtoC market (Fig. 4, Fig. S2); the trade volume has also been increasing year by year (Fig. S2). The results support the previous studies showing that an increasing number of people are purchasing meat from wildlife and livestock in online markets with the advance of internet technology (Muthukumar et al 2020; Moloney et al. 2023). Our findings also indicate the potential of online CtoC markets to support wildlife management practices (i.e., hunting and culling of sika deer and wild boar) that require continuous implementation to mitigate human–wildlife conflicts. In Japan, although efforts have been made to utilize the sika deer and wild boar culled for population control, only about 14% of the total number of harvested animals are being utilized (MAFF 2024a). Growing the online CtoC market found in this study could contribute to promoting the utilization of these WGM. Furthermore, given direct or indirect returns on sales income to hunters, the findings imply that the development of online markets with appropriate mechanisms may help to increase the motivation of hunters and enhance management efforts (MacMillan 2004; Ljung et al. 2012; Okuda et al. 2022). However, it is also important to note that the development of online markets can have negative or negligible impacts on wildlife management and biodiversity conservation (e.g., Frank et al. 2017). Therefore, appropriate regulations should be in place and strictly enforced to mitigate potential risks. In addition, effective profit distribution is essential for encouraging management efforts. For example, if intermediaries capture the highest margins, the CtoC market may fail to provide sufficient incentives for hunters and wildlife managers. Because of being beyond our scope of this study, further interdisciplinary research is needed to assess the impacts of the online CtoC market on the incentives for hunters and appropriate management, including an analysis of profit distribution along the value chain.

Despite opportunities, our findings raise concerns that the online CtoC market usage can increase risks to public health and conservation. Figure 6 presents that many offal parts of wild boar and Asiatic black bear, which requires hygiene management, were traded on the online market; additionally, wild boar offal was traded at a relatively low price. That indicates the online CtoC market makes it easy to acquire WGM offal, which is difficult to acquire in the formal food supply chain. The *Guidelines for the Hygiene Management of Wild Game Meat* recommend that offal consumption be eliminated as far as possible in Japan (Ministry of Health, Labor and Welfare (MHLW) 2021). That is because offal consumption has been associated with risks such as heavy metal contamination and parasitic infection (McCrindle et al. 2013; Sato et al. 2016; Kalinina et al. 2022). Furthermore, our findings associated with offal transactions indicate a challenge of substitutional effects. Our findings show no sales of Asiatic black bear gallbladders were observed (Table S2). This result may be attributed to strict legal regulations. In Japan, the trade of bear gallbladders is tightly controlled by law, and only individuals or organizations with official authorization are permitted to engage in such transactions (Mano and Ishii 2008; MHLW 2020). This restriction is also explicitly stated in Yahoo Auctions’ transaction guidelines, and the platform closely monitors for violations involving bear gallbladder trade. On the other hand, a large number of wild boar gallbladders, which are not regulated, were traded. This could suggest that the regulation of bear gallbladders may have unintentionally increased the trade in wild boar gallbladders. These results might support earlier conservation evidence of substitute impacts (i.e., spillover effects) caused by policy regulations (Kubo et al. 2025). Alternatively, another possible explanation for the result is that the trade in wild bear gallbladders has shifted to more covert channels, such as private messaging or the use of code words, making it harder to detect on the surface web. Our findings suggest that further development of the CtoC market requires additional monitoring effort to mitigate the risk and effective policy interventions considering potential side effects.

Moreover, the online CtoC market poses another challenge to public health. In Japan, the distribution of WGM is restricted by the *Food Sanitation Law*: WGM must be processed at licensed distribution facilities (MHLW 2021). However, the online CtoC market that we analyzed does not require presenting licenses. That makes consumers unable to accurately determine whether their WGM has been processed at a licensed facility. Similarly, the Japanese government requires traceability of properly processed WGM under “Domestic Gibier Certification System”; the traceability includes detailed information not only about the animal species and preservation methods but also about the capture date, capture method, and the responsible individuals (MAFF 2024b); whereas the clarification of traceability information is also mandated in the online CtoC market. To ensure traceability and reduce public health risks for the sustainable development of the online CtoC market, our finding, that a high volume of traded offal with low costs in the online CtoC market, suggests the necessary to mandate the clarification of traceability information for WGM trades.

The specific consumer preferences for bears are worth noting. Our results showed that trade prices for Asiatic black bears and brown bears WGM were higher than those for the other two species (Fig. 5); in particular, their Hand & Foot fetched high prices (Fig. 6). Traditionally, in Asian countries, bear hands and feet are considered a rare food with high nutritional value (Long and Li 2025) and targets of illegal trade (Lewis and Takahashi 2013), and our results revealed that the luxury trend remains on online CtoC markets. That indicates the potential conservation risks by enhancing the online CtoC market although current consumer preferences for bear products are unlikely to lead to immediate conservation threats. With the exception of some regional populations, Japanese bear populations are not considered endangered, and current levels of control culling are generally viewed as sustainable (Mano and Ishii 2008). Furthermore, the volume of online trade observed in this study was significantly lower than that of sika deer and wild boar (Fig. 4). However, in Japan, with the increase in bear conflicts with people, bears were registered in 2024 as needing tighter management, except in some areas. Given that bears in the Asian region have experienced population declines because of traditional uses (Liu et al. 2011; Davis et al. 2016), the trade volume of bear meat, including specific parts such as hands and feet, should be monitored closely for sustainable bear use and management. We believe the findings of this study provide a baseline and framework to enhance monitoring of the WGM consumption through the online markets.

Finally, our results highlight the need for further online market research on WGM. The first limitation of this study is focusing on one online market. Although approximately 10 tons of transactions were recorded annually in the market (Fig. S2), a total of 2700 tons of WGM are officially traded each year, with approximately 110 tons traded through internet transactions in Japan (MAFF 2024a). Although this is the first study showing a possibility of public health risk on online WGM transactions, the limited part of the overall online market might bias the results. Moreover, given that a lot of wildlife trade, particularly illegal ones, occurs in private messaging apps, further research should assess WGM transactions in various types of online market including multiple CtoC online markets, personal transactions, and black markets if possible. Another challenge is a possibility of misidentification. Although we systematically detected and identified animal species names in title texts, there is a possibility of misidentification if the names of animal species do not appear in the title; moreover, we could not address potential efforts by sellers to evade regulatory restrictions, such as through the use of ambiguous terms and/or codified language designed to be understood only by those within specific networks. Image identification technology and textual analysis of detailed product information would have the potential to overcome this challenge.

## Conclusion

We focused on the online CtoC market for WGM, and to our knowledge this is the first paper to identify trends in the WGM trade online in Japan. The results suggest that it has the potential to serve as a new platform for the utilization of sika deer and wild boar culled to mitigate human–wildlife conflict. We also found consumer preferences for the organs and specific parts of wild boar and black and brown bears are high. The findings imply that the online CtoC market poses public health and conservation risks. To implement the principles of “One Health,” which recognizes the connections among animals, humans, and their shared environments, our results indicate the need to monitor the trends of online WGM trades. Our findings also suggest that a dual approach is necessary; the approach involves both the establishment of guidelines for the online WGM market and the implementation of further mandatory regulations for trade monitoring, regular inspections of meat sold for possible pathogen contamination, and enforcement against violations. The approach could ensure the sustainable use of WGM in online markets while reducing the public health risks and conservation risks associated with its consumption.

## Supplementary Information

Below is the link to the electronic supplementary material.

Supplementary file1 (PDF 630 kb)

Supplementary file2 (PDF 519 kb)

## Footnotes

1 https://auctions.yahoo.co.jp/

2 https://jp.mercari.com/

3 https://jmty.jp

4 https://auctions.yahoo.co.jp/
