# Supplementary material for: Online trade of wild game meat: Implications for public health and conservation
Source: Ambio. 2025 Jul 24;55(1):68–79. doi: 10.1007/s13280-025-02221-w (PMC12672966; doi:10.1007/s13280-025-02221-w)
Supplement: Supplementary file 1 — Supplementary file1 (PDF 630 kb) [file 13280_2025_2221_MOESM1_ESM.pdf]

Supplementary Information (SI)

Endo, Mameno, Kubo in Ambio

"Online trade of wild game meat: Implications for public health and conservation"

The SI includes SI Figures and Tables:

- Fig. S1. Comparison of monthly sales before and after the amendment to the Food Sanitation Act.
- Fig. S2. Total annual sales volume of each WGM.
- Table S1. Numbers and percentages of wildlife meat traded in online sales.
- Table S2. Detail data on the trade of edible offal in wild boar and Asiatic black bears.
- Table S3. Estimation results of a generalized linear mixed model for the number of WGM parts sales.
- Table S4. Estimation results of a generalized linear mixed model for the price of WGM parts sales.

Authors and Affiliations:

Tomohiko Endo1,2, Kota Mameno3, Takahiro Kubo1,4,5*

1 Biodiversity Division, National Institute for Environmental Studies (NIES), Tsukuba, Japan

2 Institute of Livestock and Grassland Science, National Agriculture and Food Research Organization (NARO), Tsukuba, Japan

3 Research Faculty of Agriculture, Hokkaido University, Sapporo, Japan

4 Graduate School of Agriculture, Hokkaido University, Sapporo, Japan

5 School of Geography and the Environment, University of Oxford, Oxford, UK

Corresponding author:

Takahiro Kubo

Address: National Institute for Environmental Studies, 16-2 Onogawa, Tsukuba, Ibaraki 305-8506, Japan

Email: kubo.takahiro@nies.go.jp

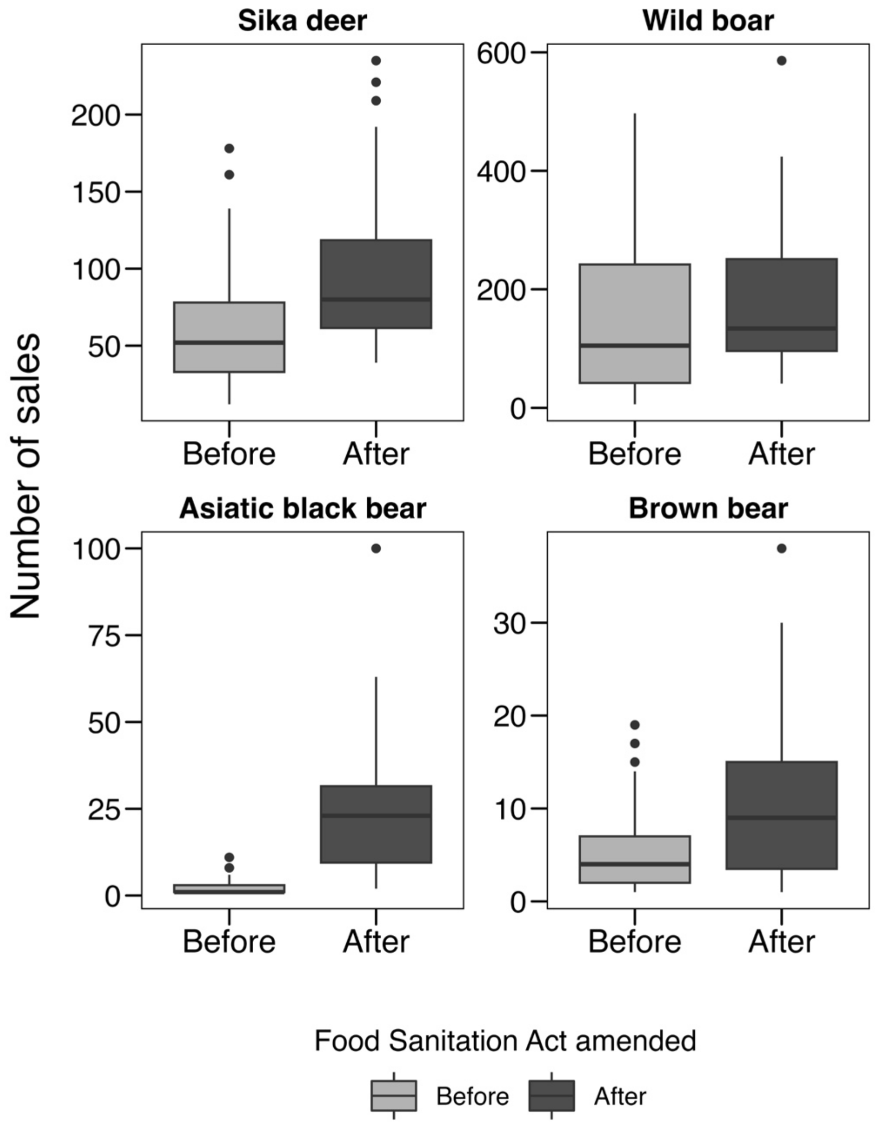

Fig. S1. Comparison of monthly sales before and after the amendment to the Food Sanitation Act.

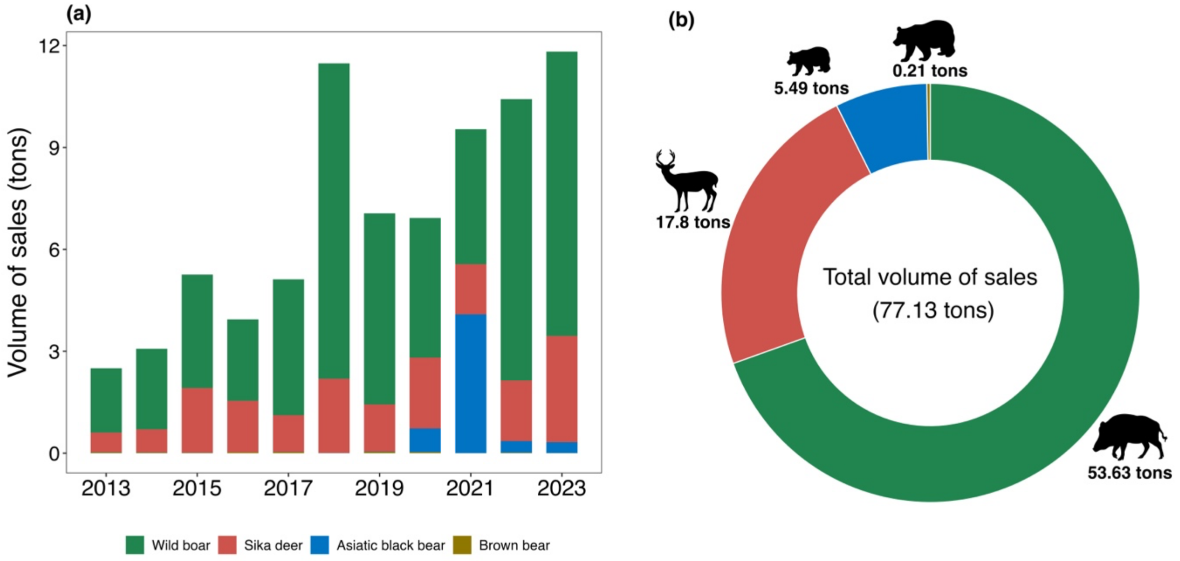

Fig. S2. Total annual sales volume of each WGM.

Note: All products with weights shown in the sales title were calculated (n = 29803).

Table S1. Numbers and percentages of wildlife meat traded in online sales.

Body part	Sika deer		Wild boar		Asiatic black bear		Brown bear	
	n	%	n	%	n	%	n	%
Head	12	0.13	61	0.28	7	0.61	3	0.39
Neck	229	2.40	688	3.19	0	0.00	0	0.00
Shank	777	8.13	159	0.74	9	0.79	8	1.04
Shoulder	456	4.77	612	2.84	32	2.79	55	7.13
Loin	2085	21.82	4334	20.10	184	16.07	175	22.70
Leg / Rump	2761	28.90	4411	20.46	187	16.33	222	28.79
Rib	1568	16.41	3259	15.11	140	12.23	105	13.62
Hand / Foot	0	0.00	0	0.00	198	17.29	88	11.41
Edible offal	399	4.18	543	2.52	132	11.53	11	1.43

Table S2. Detail data on the trade of edible offal in wild boar and Asiatic black bears.

Type of offal*	Wild boar		Asiatic black bear	
	n	%	n	%
Heart	116	16.52	38	27.74
Kidney	23	3.28	16	11.68
Liver	160	22.79	29	21.17
Stomach	16	2.28	28	20.44
Intestine	1	0.14	18	13.14
Testicle	62	8.83	5	3.65
Gallbladder	118	16.81	0	0.00
Diaphragm	17	2.42	0	0.00
Others	189	26.92	3	2.19

* Categorized and summarized based on sales description. "Others" indicates sales that were not identified (e.g. offal sets)

Table S3. Estimation results of a generalized linear mixed model for the number of WGM parts sales.

Species	Variable	Coefficient	SE	*z* value	95%CI		*P value*	
					Lower	Upper		
Sika deer	Head	-0.185	0.125	-1.475	-0.431	0.061	0.140	
	Neck	0.047	0.025	1.866	-0.002	0.096	0.062	
	Shank	0.017	0.013	1.330	-0.008	0.042	0.184	
	Shoulder	0.109	0.020	5.561	0.071	0.148	<0.001	***
	Loin	0.038	0.007	5.178	0.024	0.052	<0.001	***
	Leg & Rump	0.069	0.006	11.338	0.057	0.080	<0.001	***
	Rib	0.052	0.011	4.830	0.031	0.073	0.000	***
	Edible offal	0.037	0.019	1.966	0.000	0.074	0.049	*
Wild boar	Head	0.083	0.064	1.297	-0.042	0.208	0.195	
	Neck	-0.059	0.018	-3.368	-0.094	-0.025	<0.001	***
	Shank	0.126	0.043	2.942	0.042	0.210	0.003	**
	Shoulder	0.032	0.019	1.669	-0.006	0.070	0.095	
	Loin	0.025	0.006	3.802	0.012	0.037	<0.001	***
	Leg & Rump	0.025	0.007	3.749	0.012	0.038	<0.001	***
	Rib	0.047	0.010	4.685	0.027	0.067	<0.001	***
	Edible offal	0.124	0.021	6.024	0.084	0.164	<0.001	***
Asian black bear	Shank	-0.052	0.136	-0.380	-0.319	0.215	0.704	
	Shoulder	-0.006	0.040	-0.163	-0.084	0.071	0.871	
	Loin	0.092	0.021	4.476	0.052	0.133	<0.001	***
	Leg & Rump	0.105	0.024	4.379	0.058	0.152	<0.001	***
	Rib	0.073	0.027	2.683	0.020	0.126	0.007	**
	Hand & Foot	-0.010	0.014	-0.753	-0.037	0.017	0.452	
	Edible offal	0.091	0.020	4.565	0.052	0.130	<0.001	***
Brown bear	Head	0.337	0.143	2.361	0.057	0.618	0.018	*
	Shank	0.192	0.102	1.883	-0.008	0.392	0.060	
	Shoulder	0.139	0.038	3.630	0.064	0.215	<0.001	***
	Loin	0.051	0.024	2.138	0.004	0.098	0.033	*
	Leg & Rump	0.115	0.019	5.974	0.077	0.152	<0.001	***
	Rib	0.108	0.024	4.415	0.060	0.156	<0.001	***
	Hand & Foot	0.144	0.037	3.929	0.072	0.216	<0.001	***
	Edible offal	0.003	0.099	0.034	-0.191	0.197	0.973	

Asterisks indicate significant differences by the Wald test (*$P < 0.05$; **$P < 0.01$; ***$P < 0.001$)
95% CI: 95% confidence interval
SE: standard error

Table S4. Estimation results of a generalized linear mixed model for the price of WGM parts sales.

Species	Variable	Coefficient	SE	*z* value	95%CI		*P value*	
					Lower	Upper		
Sika deer	Head	-0.010	0.028	-0.342	-0.064	0.045	0.733	
	Neck	-0.009	0.032	-0.275	-0.072	0.054	0.784	
	Shank	0.029	0.014	2.110	0.002	0.056	0.035	*
	Shoulder	-0.032	0.020	-1.551	-0.072	0.008	0.121	
	Loin	0.031	0.036	0.854	-0.040	0.102	0.393	
	Leg & Rump	0.005	0.031	0.168	-0.056	0.067	0.867	
	Rib	-0.034	0.038	-0.882	-0.108	0.041	0.378	
	Edible offal	0.009	0.019	0.498	-0.027	0.046	0.618	
Wild boar	Head	-0.009	0.017	-0.519	-0.043	0.025	0.604	
	Neck	-0.022	0.026	-0.853	-0.073	0.029	0.394	
	Shank	-0.050	0.055	-0.906	-0.158	0.058	0.365	
	Shoulder	-0.059	0.024	-2.490	-0.105	-0.013	0.013	*
	Loin	0.070	0.026	2.647	0.018	0.121	0.008	**
	Edible offal	-0.075	0.025	-3.023	-0.123	-0.026	0.003	**
Asian black bear	Head	-0.064	0.082	-0.781	-0.224	0.096	0.435	
	Shank	0.242	0.443	0.545	-0.627	1.110	0.586	
	Shoulder	0.333	0.201	1.656	-0.061	0.726	0.098	
	Loin	-0.830	0.375	-2.214	-1.565	-0.095	0.027	*
	Leg & Rump	-0.643	0.386	-1.668	-1.399	0.113	0.095	
	Rib	-0.297	0.317	-0.939	-0.918	0.324	0.348	
	Hand & Foot	0.155	0.034	4.513	0.088	0.222	<0.001	***
	Edible offal	-0.091	0.072	-1.263	-0.233	0.050	0.207	
Brown bear	Head	0.039	0.151	0.258	-0.257	0.335	0.797	
	Shank	0.035	0.420	0.084	-0.787	0.858	0.933	
	Shoulder	-0.196	0.169	-1.159	-0.527	0.135	0.247	
	Loin	-0.083	0.406	-0.205	-0.879	0.712	0.837	
	Leg & Rump	-0.364	0.411	-0.886	-1.170	0.441	0.376	
	Rib	-0.727	0.340	-2.142	-1.393	-0.062	0.032	*
	Hand & Foot	0.701	0.084	8.397	0.538	0.865	<0.001	***
	Edible offal	-0.695	0.431	-1.611	-1.541	0.151	0.107	

Asterisks indicate significant differences by the Wald test (*$P < 0.05$; **$P < 0.01$; ***$P < 0.001$)
95% CI: 95% confidence interval
SE: standard error
