# Supplementary material for: Online trade of wild game meat: Implications for public health and conservation
Source: Ambio. 2025 Jul 24;55(1):68–79. doi: 10.1007/s13280-025-02221-w (PMC12672966; doi:10.1007/s13280-025-02221-w)
Supplement: Supplementary file 2 — Supplementary file2 (PDF 519 kb) [file 13280_2025_2221_MOESM2_ESM.pdf]

Appendix

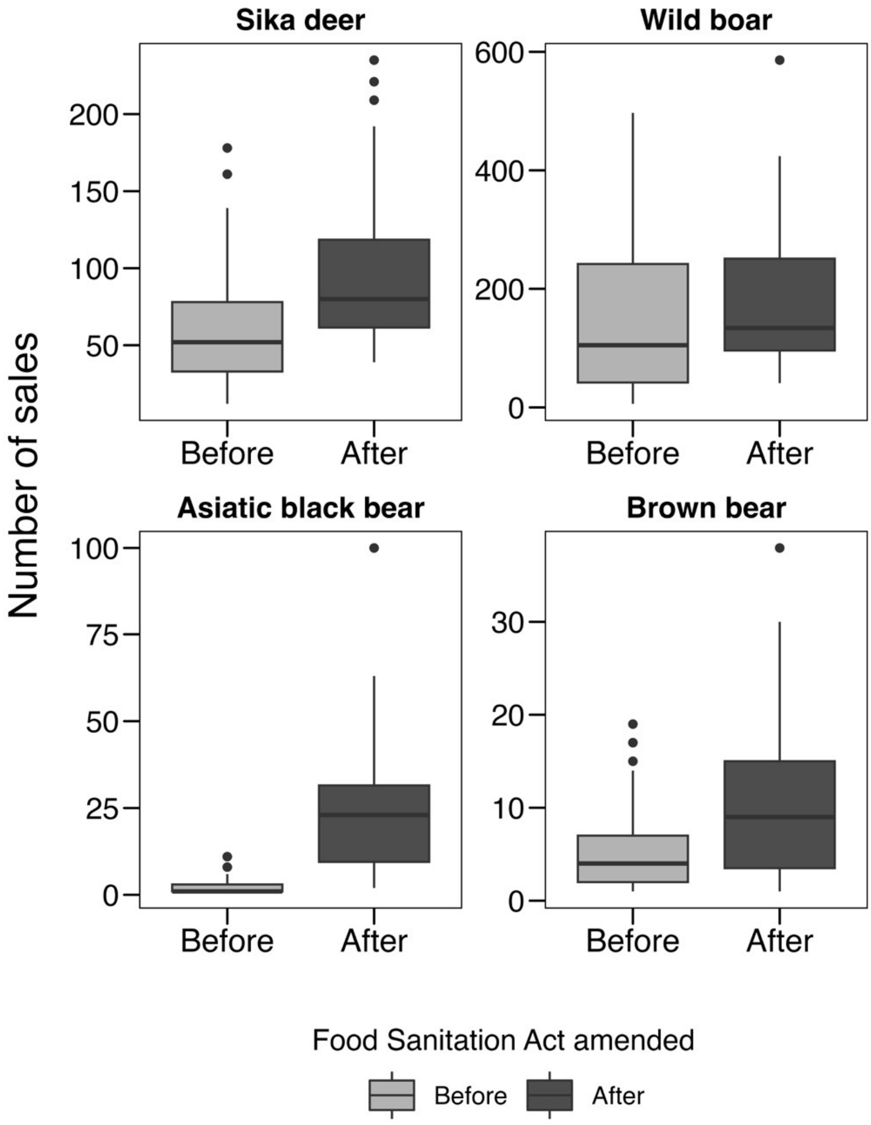

Fig. S1. Comparison of monthly sales before and after the amendment to the Food Sanitation Act.

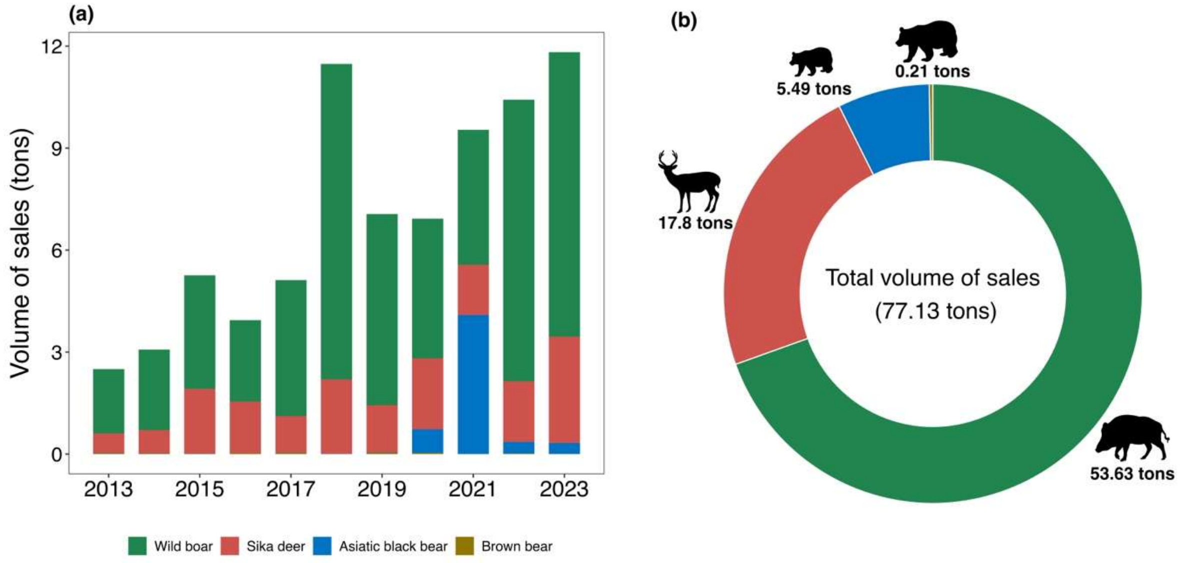

Fig. S2. Total annual sales volume of each WGM.

Note: All products with weights shown in the sales title were calculated (n = 29803).
